# Non-Pandemic *Vibrio cholerae* and *Vibrio parahaemolyticus* ST98 Recovered from Commercially Depurated Bivalves Marketed in Portugal: Whole-Genome and Phylogenomic Characterization

**DOI:** 10.3390/foods15213844

**Published:** 2026-10-27

**Authors:** Ricardo J. Figueiredo, Guilherme Moreira, Ana Machado, Eliane Silva, Adriano A. Bordalo, João R. Mesquita

**Affiliations:** 1 School of Medicine and Biomedical Sciences (ICBAS), University of Porto, 4050-313 Porto, Portugal; up202206087@up.pt (R.J.F.); ammachado@icbas.up.pt (A.M.); epsilva@icbas.up.pt (E.S.);; 2 Interdisciplinary Centre of Marine and Environmental Research (CIIMAR), University of Porto, 4450-208 Matosinhos, Portugal; 3 Research Center in Biodiversity and Genetic Resources (CIBIO/InBIO), University of Porto, 4485-661 Vairão, Portugal; 4 BIOPOLIS Program in Genomics, Biodiversity and Land Planning, CIBIO, Campus de Vairão, 4485-661 Vairão, Portugal; 5 Department of Chemistry and Biochemistry, Faculty of Sciences, LAQV, REQUIMTE, University of Porto, 4050-313 Porto, Portugal

**Keywords:** *Vibrio cholerae*, *Vibrio parahaemolyticus*, bivalve mollusks, whole-genome sequencing, seafood safety, genomic surveillance

## Abstract

*Vibrio cholerae* and *Vibrio parahaemolyticus* are naturally occurring aquatic bacteria of public-health relevance that may contaminate seafood, including bivalve mollusks. This study characterized two *Vibrio* isolates recovered from commercially depurated mussels (*Mytilus* spp.) and clams (*Spisula solida*) marketed at retail in Portugal. Species identification, antimicrobial susceptibility testing, Oxford Nanopore whole-genome sequencing (WGS), average nucleotide identity analysis, antimicrobial resistance and virulence profiling, genoserotyping, prophage prediction, and core-genome phylogenomic analysis were performed. WGS confirmed the mussel-derived isolate as *V. cholerae* and the clam-derived isolate as *V. parahaemolyticus*. Phenotypic testing demonstrated meropenem resistance in the *V. cholerae* isolate, whereas several additional antimicrobial MICs could not be categorically interpreted because of panel concentration limits or the absence of *Vibrio*-specific clinical breakpoints. The *V. cholerae* isolate lacked *ctxA*, *ctxB*, the toxin-coregulated pilus cluster, *ace*, and *zot*, while its O1/O139 serogroup status remained unresolved because an O1-associated marker was absent and a complete but non-exact *wbfZ*-like sequence was detected. Its genome nevertheless contained an extensive Type VI secretion system repertoire together with several hemolysin- and toxin-associated determinants. Phylogenomically, the isolate was most closely related to the environmental strain RFB16 and was distinct from the pandemic reference lineages included in the analysis. The *V. parahaemolyticus* isolate was assigned to ST98 and OL4 and carried a divergent KL55-like K-antigen locus. Its virulence-associated repertoire was dominated by a well-conserved Type III secretion system 1, together with adhesion-, motility-, and hemolysin-associated determinants, whereas the major enteropathogenic markers *tdh* and *trh* were absent. It grouped most closely with strain vp-HL-202008, although this relationship received moderate phylogenetic support. Resistance-associated determinants were predominantly chromosomal, and prophage analysis identified two putative regions in *V. cholerae* and seven in *V. parahaemolyticus*. Overall, these findings characterize two *Vibrio* isolates recovered from commercially depurated bivalves marketed in Portugal. Their detection does not demonstrate depuration failure or an immediate consumer-health risk but highlights that conventional indicator-based compliance does not necessarily exclude autochthonous *Vibrio* spp. and supports the value of genomic surveillance in seafood safety.

## 1. Introduction

*Vibrios* are Gram-negative bacteria belonging to the family *Vibrionaceae*, widely distributed in aquatic environments [1]. The genus *Vibrio* comprises more than 160 described species, with its taxonomy continuously evolving through advances in molecular and genomic approaches [2,3]. Commonly, *Vibrio* spp. possess two chromosomes of different sizes, with the smaller chromosome being reported as more flexible than the larger one during the evolution of *vibrios* [4].

Ecologically, these bacteria are naturally associated with aquatic environments and occur across a broad range of salinities, including fresh, brackish, and marine environments [1].

In these habitats, they are frequently associated with marine life, particularly sea fish, mollusks, and crustaceans [2].

However, the consumption of these marine products, particularly bivalve mollusks such as mussels and clams, may represent a food safety and public health concern when potentially pathogenic *Vibrio* spp. are present, especially when these products are consumed raw or undercooked or are subjected to inadequate refrigeration and improper handling or storage conditions that may favor bacterial survival and proliferation [5]. Due to their filter-feeding nature, bivalves process large volumes of the water column to obtain nutrients and may accumulate pathogenic *Vibrio* species in their tissues and digestive tract [6]. Consequently, if these bivalves are harvested from areas with elevated bacterial loads and subsequently consumed raw, undercooked, or poorly preserved, they may act as primary vectors for human foodborne infections [7]. Among the *Vibrio* species associated with seafood-borne disease, *Vibrio parahaemolyticus* and *Vibrio cholerae* are of particular public health relevance [8,9]. *Vibrio parahaemolyticus* is widely recognized as the leading cause of acute seafood-borne gastroenteritis, with infections ranging from self-limiting mild gastrointestinal illness to severe complications in susceptible individuals, like hypotension and shock, which, if untreated, can lead to fatal outcomes [10]. The global dissemination of *V. parahaemolyticus*, including the highly virulent pandemic O3:K6 clone, has been exacerbated by climate change [11]. Rising sea surface temperatures may facilitate the geographical expansion of this pathogen into historically colder temperate regions, triggering an increase in foodborne outbreaks across European coastal waters [12].

In contrast, *Vibrio cholerae* is the causative agent of cholera, a severe diarrheal disease [13].

Epidemic cholera is primarily associated with toxigenic strains belonging to serogroups O1 and O139 and remains an important global public health problem, particularly in regions with inadequate water, sanitation, and hygiene infrastructure [14]. To address this burden, the Global Task Force on Cholera Control (GTFCC) launched the comprehensive strategy “Ending Cholera: A Global Roadmap to 2030”, aiming to reduce cholera mortality and strengthen prevention and control strategies in affected countries [15]. Beyond epidemic O1 and O139 lineages, non-O1/non-O139 *V. cholerae* strains are also frequently detected in aquatic and coastal environments and have been associated with seafood-borne and opportunistic infections [8,16].

The presence of these pathogens in marine environments is therefore relevant to seafood safety, particularly in countries with high seafood consumption [1,5]. In coastal countries like Portugal, fish and seafood represent an important source of nutrients and contribute to economic development [17]. Given the prominent role of mussels and clams in Portuguese seafood consumption, characterizing *Vibrio* spp. associated with commercially available bivalves can contribute to a better understanding of their potential relevance for food safety [18,19].

Recent European data further support the relevance of these pathogens in seafood, with *V. parahaemolyticus* detected in approximately 19.6% of seafood samples and in 27.8% of bivalve mollusks, while non-choleragenic *V. cholerae* showed an estimated pooled prevalence of 4.1% across available European studies [5]. In Portugal, recent surveillance of seafood marketed for human consumption detected non-O1/non-O139 *V. cholerae* in 43 of 129 samples (33.3%), with WGS revealing genetically diverse isolates carrying virulence-, fitness-, and antimicrobial-resistance-associated determinants [20]. These findings highlight the continued value of combining conventional isolation and molecular detection with genomic characterization to better define the diversity and potential food-safety relevance of *Vibrio* populations circulating in seafood.

Despite these concerns, establishing official microbiological criteria for *Vibrio* species in seafood remains a complex challenge. Under European legislation, the microbiological criteria regarding the food safety of raw bivalve mollusks have historically relied on fecal indicators, such as *Escherichia coli* quantitative monitoring and the absence of *Salmonella* [21]. Consequently, bivalves harvested from Class B and Class C production areas, which correspond to areas with progressively higher levels of fecal contamination based on *Escherichia coli* criteria, require post-harvest treatment, such as depuration or relaying, before being placed on the market for human consumption. However, while these post-harvest purification treatments are highly effective at reducing enteric bacteria, their efficiency in eliminating naturally occurring marine bacteria like *Vibrio* species can be limited and may vary according to bacterial, host, and process-related factors [22]. The foundational Regulation (EC) No 2073/2005 does not establish specific microbiological limits for *Vibrio* pathogens, stating the need for further risk assessment protocols and the development of standardized analytical methods before such criteria can be effectively implemented [21]. This highlights a critical gap in routine monitoring, as compliance with traditional bacterial indicators does not necessarily guarantee the absence of potentially pathogenic *Vibrio* strains in bivalve matrices.

Whole-genome sequencing (WGS) has become an essential tool for the characterization of foodborne and environmental bacteria, enabling accurate species identification, detection of antimicrobial resistance- and virulence-associated determinants, and comparative phylogenomic analysis [23,24]. Beyond population-level surveillance, detailed genomic investigation of individual isolates can provide valuable insights into their genetic characteristics, particularly when conventional phenotypic identification is inconclusive or when specific antimicrobial susceptibility and virulence-associated profiles warrant further investigation. This is particularly relevant for *Vibrio* spp. recovered from commercially depurated bivalves, as routine microbiological monitoring does not specifically target these naturally occurring aquatic bacteria. Moreover, integrating phenotypic and genomic approaches offers an opportunity to establish comprehensive analytical frameworks that may be adapted for future investigations involving larger isolate collections, different seafood matrices, and broader geographical and temporal sampling.

Against this background, the present study aimed to provide a retrospective phenotypic and genomic characterization of two archived *Vibrio* isolates recovered in 2016 from independent commercial batches of mussels (*Mytilus* spp.) and clams (*Spisula solida*) marketed in Portugal following commercial depuration. These isolates were selected for detailed investigation because their identification could not be conclusively resolved using conventional automated methods, and their recovery from distinct commercially available bivalve matrices provided an opportunity to investigate genomic characteristics potentially relevant to seafood microbiology and food safety. By integrating antimicrobial susceptibility testing with long-read WGS, we investigated their taxonomic identity, antimicrobial resistance- and virulence-associated determinants, antigen-associated loci, prophage content, and phylogenomic relationships. In addition to generating publicly accessible genomic reference data for these specific strains, the study illustrates the application of an integrated analytical workflow that could serve as a methodological foundation for future genomic surveillance studies of seafood-associated *Vibrio* spp., subject to further evaluation and standardization. Importantly, given the limited number of isolates and their recovery within a single sampling year, the present investigation was not intended to assess prevalence, population-level diversity, temporal trends, or the circulation of particular lineages. Instead, its contribution lies in the detailed characterization of two archived isolates and in demonstrating the potential applicability of a comprehensive, adaptable approach to future investigations involving more representative sampling strategies.

## 2. Materials and Methods

### 2.1. Sample Collection

The isolates analyzed in this study belonged to a previously established collection of bacteria recovered from retail bivalve mollusks purchased in Portugal since January 2016. For the present study, two independent retail bivalve samples were considered, comprising one mussel (*Mytilus* spp.) sample and one clam (*Spisula solida*) sample. Both samples were purchased and processed in 2016 and corresponded to independent commercial batches of approximately 1 kg each. The mussel sample originated from Spanish aquaculture, whereas the clam sample was harvested from the Northeast Atlantic.

For the mussel isolate, fresh, chilled mussel samples (*Mytilus* spp.) were purchased from a commercial retail outlet located in Northern Portugal. The mussels analyzed in this study originated from a Class B production area, defined under European legislation as an area in which *Escherichia coli* levels in live bivalve mollusks do not exceed 4600 CFU/MPN per 100 g of flesh and intravalvular liquid in 90% of samples and 46,000 per 100 g in the remaining 10%. Accordingly, bivalves from Class B areas require post-harvest treatment, such as depuration or relaying, before being placed on the market for human consumption. The samples analyzed here were obtained as a retail product following commercial depuration.

The country in which depuration was performed was not recorded in the available sample documentation. Both products were subsequently marketed for human consumption in Portugal.

The product was therefore considered compliant with the microbiological criteria required for sale and consumption. Following purchase, the samples were transported to the laboratory in insulated containers maintained at approximately 4 °C to preserve microbiological integrity.

Similarly, the clam (*Spisula solida*) isolate originated from a Class B harvesting area and had undergone commercial depuration.

The handling and transport processes were identical to those described for the mussels. For microbiological processing, approximately 25 g of pooled soft tissues and intravalvular fluid were obtained from each of the two commercial samples. The exact number of individual mussels or clams contributing to each pooled 25 g analytical portion was not recorded.

### 2.2. Sample Preparation

Bivalve samples were processed under aseptic conditions following a previously described method, with minor modifications [25,26]. Briefly, approximately 25 g of pooled soft tissues and intravalvular fluid from multiple individuals belonging to the same commercial bivalve sample were transferred to a sterile homogenization bag and combined with 225 mL of alkaline peptone water (APW) corresponding to a sample-to-diluent ratio of 1:9 and yielding a tenfold initial suspension. Thus, approximately 25 g of material from each independent bivalve sample was subjected to microbiological analysis. The mixture was then homogenized for 2 min at maximum speed using a peristaltic stomacher and subsequently used for microbiological analysis.

### 2.3. Bacterial Identification and Antimicrobial Susceptibility Testing (AST)

Following homogenization, samples were incubated for 18 h at 35 C° for the selective enrichment of *Vibrio* spp. Following incubation, enriched cultures were streaked onto CHROMagar^TM^ *Vibrio* agar, and colonies exhibiting morphology compatible with *Vibrio* spp. were selected and subcultured to obtain pure cultures. Blue colonies were considered presumptive *V. cholerae*, while pink colonies were considered presumptive *V. parahaemolyticus*. The isolates were incubated at 37 °C for 24 h and subsequently cryopreserved at −80 °C in Luria–Bertani broth supplemented with 25% glycerol until further analysis. After recovery from frozen stocks, isolate purity was confirmed by subculture, and bacterial morphology and Gram-negative status were assessed by Gram staining [27]. Automated identification and antimicrobial susceptibility testing were performed using the MicroScan WalkAway Plus system equipped with Neg-Urine Combo 98 panels (Beckman Coulter, Tokyo, Japan), in accordance with the manufacturer’s instructions. The panel was used to determine the minimum inhibitory concentrations (MICs) of β-lactams, including ampicillin, amoxicillin–clavulanic acid, piperacillin–tazobactam, aztreonam, cefuroxime, cefoxitin, cefotaxime, cefotaxime–clavulanic acid, ceftazidime, ceftazidime–clavulanic acid, cefepime, ertapenem, imipenem, and meropenem; aminoglycosides, including amikacin, gentamicin, and tobramycin; quinolones and fluoroquinolones, including nalidixic acid, ciprofloxacin, levofloxacin, and norfloxacin; as well as colistin, fosfomycin, nitrofurantoin, and trimethoprim–sulfamethoxazole. MIC values were interpreted according to the EUCAST v.16.1 breakpoint criteria adopted for the study [27]. The isolates selected for further characterization were subsequently subjected to WGS.

### 2.4. Genomic DNA Extraction

For genomic DNA preparation, each isolate was cultured in brain heart infusion broth (BHI; Liofilchem, Roseto degli Abruzzi, Italy) at 37 °C for 24 h. A 5 mL aliquot of each culture was subsequently centrifuged at 13,000× *g* for 10 min. Following centrifugation, 4 mL of supernatant was removed, and the bacterial pellet resuspended in the remaining culture medium was transferred to a 2 mL microcentrifuge tube containing 0.1 mm Precellys beads that had been acid-washed with 1 M HCl and subsequently rinsed with sterile water. Mechanical cell disruption was performed using a TissueLyser (Qiagen, Hilden, Germany) at 15 Hz for 3 min. Genomic DNA was then purified using the Mag-Bind^®^ Universal Pathogen Core Kit according to the manufacturer’s instructions. DNA concentration was determined fluorometrically using a Qubit™ instrument and the Qubit™ 1× dsDNA High-Sensitivity Assay Kit (Thermo Fisher Scientific, Waltham, MA, USA). Samples were submitted for WGS at concentrations meeting the sequencing provider’s recommended input requirements (50 ng/µL for purified bacterial genomic DNA).

### 2.5. Bacterial Whole-Genome Sequencing

Genomic DNA samples with concentrations of approximately 50 ng/µL were submitted to Plasmidsaurus (London, UK) for WGS. Long-read sequencing was performed using Oxford Nanopore Technologies (ONT; Oxford, UK) on a PromethION 24 instrument fitted with an R10.4.1 flow cell. Sequencing libraries were generated with the Native Barcoding Kit 96 V14 (SQK-NBD114.96; ONT), following the manufacturer’s recommendations.

Raw nanopore signal data were basecalled in super-accurate mode using Dorado for PromethION v7.4.12, with reads below a Q-score of 10 excluded [28]. Preliminary demultiplexing and removal of barcode and adapter sequences were performed using MinKNOW v6.0.4. Read-quality metrics were subsequently evaluated with NanoPlot v1.43.0. Any remaining adapter or barcode sequences were removed using Porechop v0.2.4, after which NanoFilt v2.8.0 was applied to retain reads with a mean quality score of at least 10 [29].

### 2.6. Whole-Genome Analysis

Raw Oxford Nanopore long reads were initially filtered using Filtlong v0.2.1 to discard the lowest-quality 5% of reads. Following a preliminary structural assembly of 250 Mb of downsampled sequencing data using Miniasm v0.3, datasets were further refined with Filtlong (--mean_q_weight 10) to achieve approximately 200× high-quality genome coverage per isolate. *De novo* assembly was performed using Flye v2.9.1, followed by polishing with Medaka v1.8.0. Final assembly metrics and contiguity metrics were evaluated using QUAST v5.3.0 [30,31].

Taxonomic classification was verified via Average Nucleotide Identity using pyANI v0.2.12 [32], while genome completeness and contamination levels were evaluated with CheckM v1.2.5 [33] prior to structural and functional annotation using the comprehensive Bakta v1.12.0 database [34]. Sequence types were assigned in silico utilizing MLST v2.0 [35], while plasmid replicons and mobility-associated elements were investigated using PlasmidFinder v2.1 and the MOB-scan server, respectively.

To characterize the resistome and virulome, genome assemblies were screened using ABRicate v1.0.0 against the ResFinder, NCBI, CARD, MEGARes, and ARG-ANNOT databases for antimicrobial resistance genes, and VFDB for virulence factors [36,37,38]. Additionally, prophages and viral sequences were mapped using PHASTER [39,40]. Hits were retained when they showed ≥80% nucleotide identity and ≥80% sequence coverage relative to the corresponding reference sequence, consistent with the default ABRicate filtering thresholds.

The predicted human pathogenic potential of each genome was additionally assessed using PathogenFinder2 v0.6.0, applying the default classification threshold of 0.5.

Reference genomes were retrieved from GenBank by filtering for the target species and complete genome assemblies. Candidate genomes were then manually reviewed at the strain level, and a curated subset was selected based on their epidemiological, ecological, or phylogenetic relevance for comparison with the study isolates, including well-characterized reference and clinically or environmentally relevant strains. The optimal nucleotide substitution models were selected by the Bayesian Information Criterion: TIM2+F+I+R4 for *V. cholerae* and GTR+F+I+R3 for *V. parahaemolyticus* [41,42]. Trees and associated isolation metadata were subsequently visualized using iTOL v6 [43].

The draft genome assemblies were deposited in the DDBJ/ENA/GenBank databases. The *V. cholerae* genome (BioProject PRJNA1470181; BioSample SAMN60399037) is available under WGS accession JCASQS000000000 (version JCASQS010000000), while the *V. parahaemolyticus* genome (BioProject PRJNA1470184; BioSample SAMN60399286) is accessible under WGS accession JCASQT000000000 (version JCASQT010000000).

In silico serogrouping and serotyping were performed using the assembled genome sequences in FASTA format. The *Vibrio parahaemolyticus* isolate was genoserotyped using Kaptive v2.0.6 [44] with the VibrioTyper reference databases [45], which identify the genomic loci associated with the O and K surface antigens and assign the corresponding O:K genotype based on comparison with reference loci. The *Vibrio cholerae* isolate was analyzed using VISTA v3.0.1 [46], with serogroup assignment based on the detection and sequence similarity of specific O-antigen-associated markers. Results were interpreted according to locus coverage, nucleotide identity, gene completeness, and the presence of disrupted or incomplete marker sequences.

## 3. Results

### 3.1. Phenotypic Identification and Antimicrobial Susceptibility Testing

The two Gram-negative isolates recovered from mussel and clam samples could not be conclusively identified at the species level using the Neg-Urine Combo 98 panel on the MicroScan WalkAway Plus system (Beckman Coulter, Tokyo, Japan), as the system did not provide species-level identifications with sufficient confidence. Species identity was subsequently confirmed by WGS.

The same panel was used to determine the MICs of the antimicrobial agents tested against both isolates. The resulting antimicrobial susceptibility profiles are presented in Table 1.

According to the EUCAST v.16.1 interpretive criteria applied in this study, the mussel-derived *Vibrio cholerae* isolate (JCASQS010000000) was classified as resistant to meropenem, with an MIC of 4 mg/L, whereas the clam-derived *Vibrio parahaemolyticus* isolate (JCASQT010000000) was susceptible to meropenem, with an MIC of ≤0.12 mg/L. Both isolates were susceptible to ceftazidime.

For cefotaxime, trimethoprim–sulfamethoxazole, and piperacillin–tazobactam, the MIC values reported by the automated panel were censored and did not allow categorical interpretation relative to the applicable EUCAST breakpoints. These results were therefore classified as not interpretable (NI). For several other antimicrobial agents, including colistin and tobramycin, no *Vibrio*-specific EUCAST clinical breakpoints were available; consequently, the corresponding MICs are reported without categorical susceptibility interpretation.

The same approach was applied to fosfomycin. The fosfomycin MIC was 32 mg/L for the *V. cholerae* isolate and ≤8 mg/L for the *V. parahaemolyticus* isolate. However, because no validated *Vibrio*-specific EUCAST clinical breakpoint is available for fosfomycin, these values were reported descriptively and were not assigned to susceptible or resistant categories. Similarly, the observed differences in colistin MICs (4 and ≤2 mg/L for *V. cholerae* and *V. parahaemolyticus*, respectively) and tobramycin MICs (≤2 and 4 mg/L, respectively) should be interpreted quantitatively rather than as categorical evidence of susceptibility or resistance.

### 3.2. Genome Assembly, Taxonomic Classification and Genoserotyping

#### 3.2.1. *Vibrio cholerae* JCASQS010000000

The isolate recovered from commercially depurated mussels (*Mytilus* spp.) was confirmed as *Vibrio cholerae* through WGS and Average Nucleotide Identity analysis. The genome, deposited under accession number JCASQS010000000, displayed an ANI value of approximately 98.08% relative to the reference genome GCA_008369605.1, supporting its assignment to *V. cholerae* at the species level. This genome-based classification resolved the inconclusive phenotypic identification obtained with the MicroScan WalkAway Plus system.

The de novo assembly resulted in two chromosomes, consistent with the bipartite chromosome architecture characteristic of *Vibrio* species. Assembly statistics and contiguity were evaluated using QUAST v5.3.0. The larger contig had a total length of 3,013,752 bp, while the smaller contig was 971,381 bp, with an overall genome GC content of 47.55%. QUAST analysis also reported an N50 of 3,013,752 bp, an N90 of 971,381 bp, an auN of 2,515,921.6, and L50 and L90 values of 1 and 2, respectively. The assembly contained no ambiguous bases, with 0.00 N’s per 100 kbp. Genome quality and integrity were further assessed using CheckM v1.2.5, which revealed a genome completeness of 99.98% and a contamination value of 0.1%.

In silico screening using VISTA did not provide a definitive serogroup assignment for the *Vibrio cholerae* isolate. The O1-associated marker *rfb*V was not detected. Conversely, a complete and apparently non-disrupted, but not exact, match to the O139-associated marker *wbf*Z was identified on contig 1, displaying 96.42% identity to the reference sequence. Therefore, the isolate could not be conclusively assigned to either the O1 or O139 serogroup based on the VISTA output.

Among the 19 individual virulence-associated genes investigated, six were classified as present: *chx*A, *hap*A, *hly*A, *mak*A, *rpo*S, and *tox*R. The *chx*A toxin gene showed an exact, complete, and non-disrupted match with 100% identity. Complete and non-disrupted matches were also obtained for *mak*A (99.55%), *tox*R (98.94%), *rpo*S (98.91%), *hap*A (98.25%), and *hly*A (97.17%).

Three additional virulence-associated genes were classified as incomplete. Only a short disrupted fragment of *nan*H was identified, providing insufficient evidence for the presence of a complete neuraminidase gene. The adhesion-associated gene *omp*U was represented by two partial matches and was consequently classified as incomplete. A high-similarity match to *vas*X was also detected, although the sequence was classified as disrupted and therefore incomplete.

The cholera toxin genes *ctx*A and *ctx*B were not detected. The additional toxin-associated genes *ace*, *stn*, and *zot* were also absent, as were the colonization-associated genes *acf*A, *acf*B, *acf*C, and *acf*D, and the mucinase-associated gene *tag*A.

Overall, genome-based analyses confirmed the mussel-derived isolate (JCASQS010000000) as *Vibrio cholerae*.

**Figure 1:**
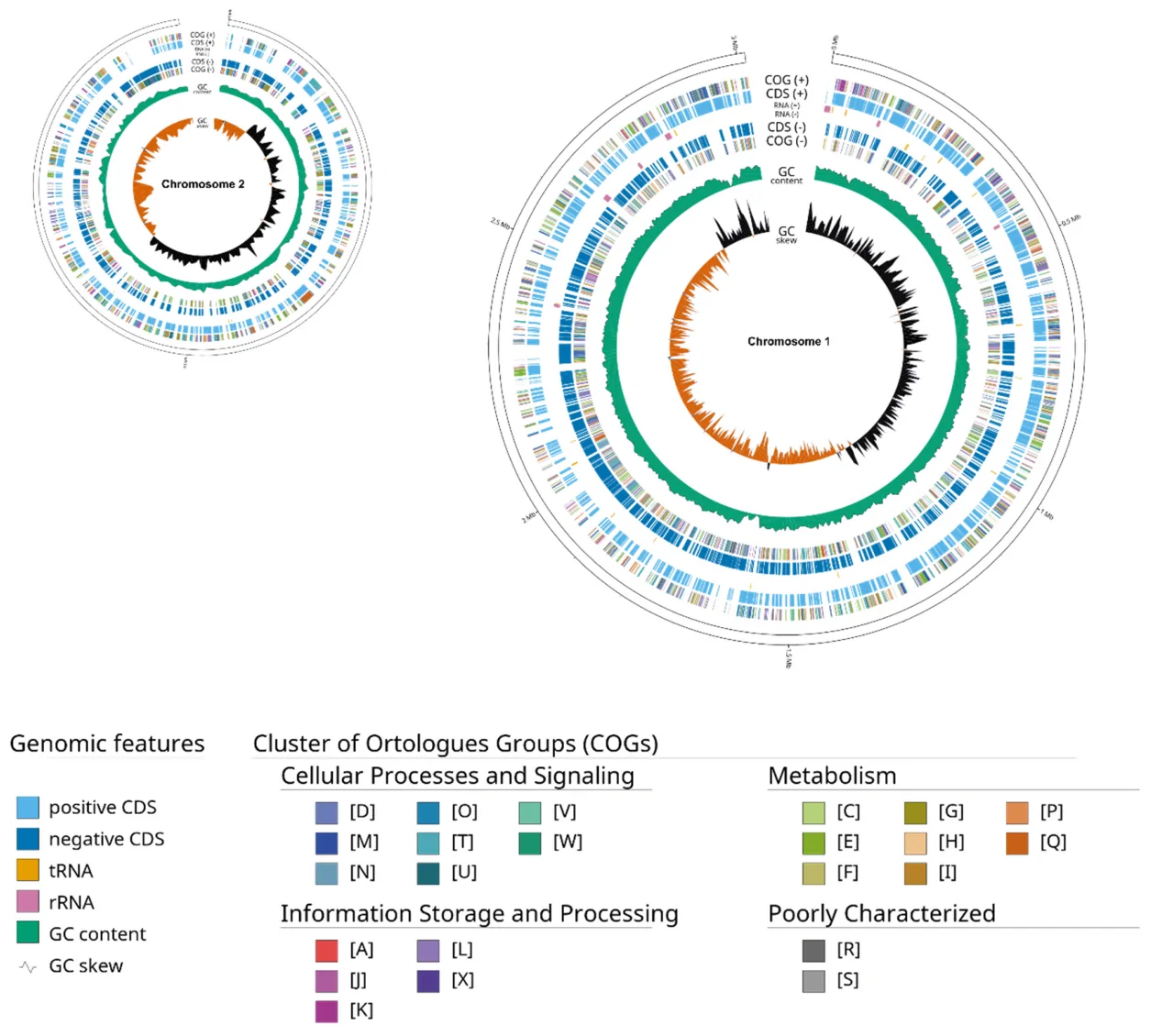
Circular representation of the *Vibrio cholerae* genome assembly. The complete genome consists of two chromosomes, corresponding to the expected bipartite genome architecture of *Vibrio cholerae*. Moving from the outermost ring to the innermost, the concentric circles represent: (1) Clusters of Orthologous Groups (COG) functional classification of predicted protein-coding sequences (CDS), with genes color-coded according to their assigned functional category; (2) predicted CDS on the forward strand; (3) predicted CDS on the reverse strand; (4) GC content, plotted relative to the average genomic GC content, with green indicating values above the genomic average and black indicating values below the average; and (5) GC skew [(G − C)/(G + C)], with orange indicating positive values and black indicating negative values.

An overview of its genomic organization and functional annotation is presented in Figure 1.

#### 3.2.2. *Vibrio parahaemolyticus* JCASQT010000000

The isolate recovered from commercially depurated clams (*Spisula solida*) was confirmed as *Vibrio parahaemolyticus* through WGS and Average Nucleotide Identity analysis. The genome, deposited under accession number JCASQT010000000, exhibited an ANI value of approximately 98.52% relative to the reference genome GCA_000196095.1, with aligned genomic fractions ranging from 90.58% to 92.24%. These results supported its taxonomic assignment to *V. parahaemolyticus* at the species level and resolved the inconclusive phenotypic identification obtained using the MicroScan WalkAway Plus system. The *de novo* assembly of the *Vibrio parahaemolyticus* isolate consisted of three contigs, with a total length of 5,262,196 bp. The two largest contigs measured 3,450,421 bp and 1,803,584 bp, respectively, and were consistent with the bipartite chromosome architecture of *Vibrio* species, while an additional small contig of 8191 bp was also assembled. The assembly had an overall GC content of 45.19% and contained no ambiguous bases, with 0.00 N’s per 100 kbp. QUAST v5.3.0 further reported an N50 of 3,450,421 bp, an N90 of 1,803,584 bp, an auN of 2,880,620.1, and L50 and L90 values of 1 and 2, respectively. CheckM analysis indicated a low contamination level of approximately 0.1% in both isolates.

The isolate was further characterized in silico through multilocus sequence typing, O- and K-antigen genotyping, virulence-gene screening, antimicrobial resistance profiling, and plasmid-associated sequence analysis. Isolate JCASQT010000000 was confirmed as *Vibrio parahaemolyticus* and assigned to sequence type ST98. The allelic profile comprised *dnaE*-14, *gyrB*-30, *recA*-49, *dtdS*-11, *pntA*-27, *pyrC*-11, and *tnaA*-13. All seven housekeeping loci showed exact matches to complete and intact alleles deposited in the PubMLST database, supporting an unambiguous ST assignment. In silico O- and K-antigen locus typing assigned the isolate to O-antigen locus OL4. KL55 was identified as the closest K-antigen locus match, with 100% locus coverage and 96.97% nucleotide identity; however, the assignment was classified as low confidence. Of the 26 genes expected in the KL55 reference locus, 25 were detected, while *KL55_04* was absent. In addition, the locus contained *KL69_04* and *KL4_06*, showing 92.11% and 100% nucleotide identity, respectively. These differences suggest a divergent or potentially mosaic KL55-related locus. The isolate was therefore conservatively described as OL4 with a KL55-like K antigen locus rather than being unequivocally classified as O4 serotype.

**Figure 2:**
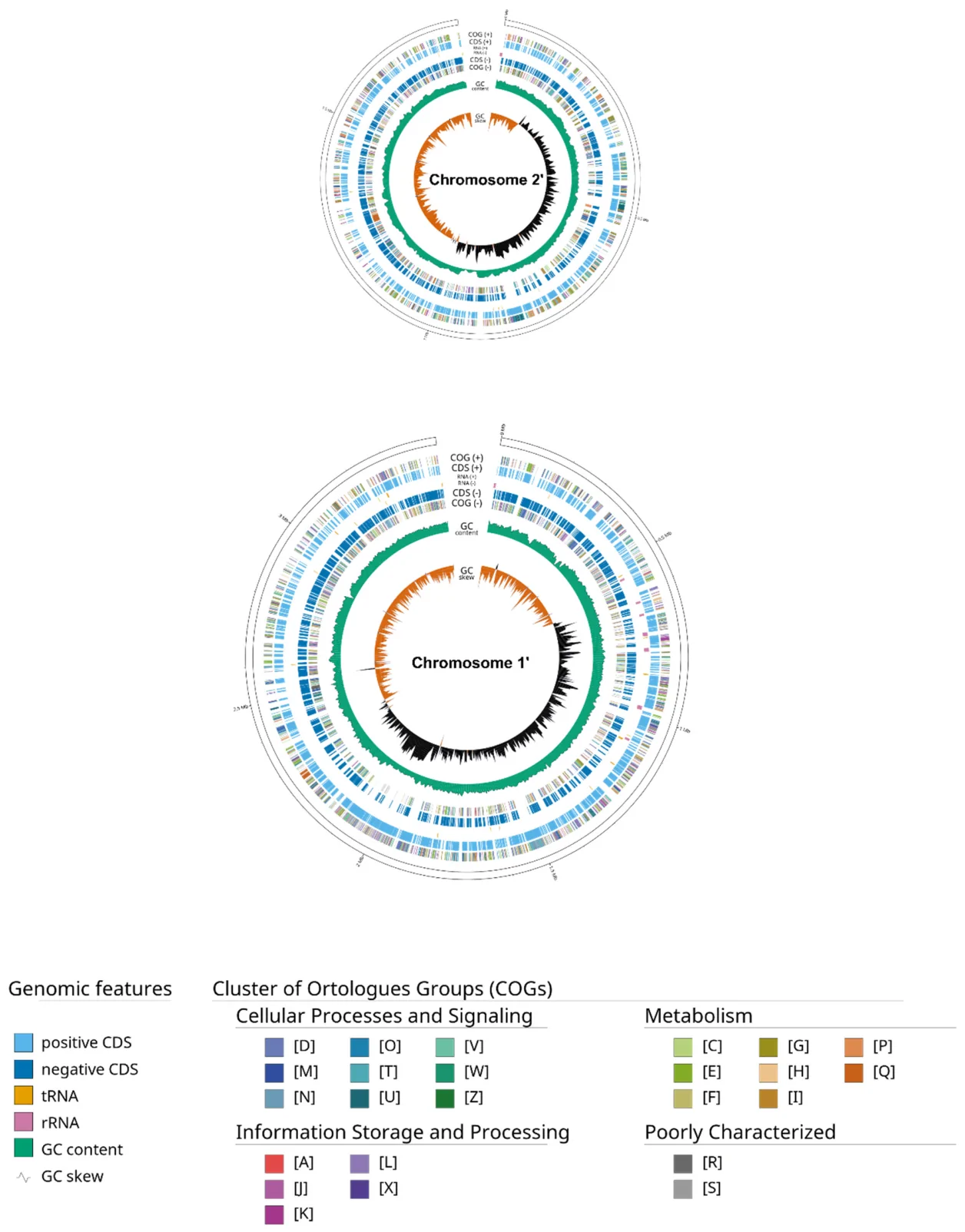
Circular representation of the *Vibrio parahaemolyticus* genome assembly. The complete genome consists of two chromosomes, consistent with the expected bipartite genome architecture of *Vibrio parahaemolyticus*. Moving from the outermost ring to the innermost, the concentric circles represent the following genomic features: (1) Clusters of Orthologous Groups (COG) functional classification of predicted protein-coding sequences (CDS), with genes color-coded according to their assigned functional category; (2) predicted CDS on the forward strand; (3) predicted CDS on the reverse strand; (4) GC content, plotted relative to the average genomic GC content, with green indicating values above the genomic average and black indicating values below the average; and (5) GC skew [(G − C)/(G + C)], with orange indicating positive values and black indicating negative values.

An overview of its genomic organization and functional annotation is presented in Figure 2.

### 3.3. Antimicrobial Resistance Profiles

#### 3.3.1. *Vibrio cholerae* Isolate (JCASQS010000000)

To investigate the antimicrobial resistance determinants of the *Vibrio cholerae* isolate and their potential relationship with the phenotypic antimicrobial susceptibility profile, the genome assembly was screened against several antimicrobial resistance databases using ABRicate, namely CARD, ResFinder, ARG-ANNOT, MEGARes, and the NCBI Bacterial Antimicrobial Resistance Reference Gene Database.

The identified resistance-associated determinants are displayed in Table 2.

The *in silico* analysis identified 11 resistance-associated genes, all allocated on the larger chromosome (3,013,752 bps). The detected determinants were mainly associated with multidrug efflux, regulation or efflux systems, and reduced susceptibility to cationic antimicrobial compounds.

Overall, the identified determinants showed nearly complete gene coverage, ranging from 99.68% to 100%, with sequence identity values between 82.05% and 99.90%. Most hits displayed sequence identity over 97%.

#### 3.3.2. *Vibrio parahaemolyticus* Isolate (JCASQT010000000)

Similarly, the *Vibrio parahaemolyticus* genome was screened against CARD, ResFinder, ARG-ANNOT, MEGARes, and the NCBI Bacterial Antimicrobial Resistance Reference Gene Database using ABRicate. The identified resistance-associated determinants are presented in Table 3.

In silico screening identified a *bla*CARB-like β-lactamase gene on the putative smaller chromosome (1,803,584 bp) and two tetracycline resistance-associated determinants, tet(35) and tet(34)-like. The detected determinants showed high coverage values, ranging from 99.28% to 100%, with sequence identity values ranging from 83.05% to 99.77%.

### 3.4. Virulence-Associated Genomic Profiles and Pathogenicity Prediction

#### 3.4.1. *Vibrio cholerae* Isolate (JCASQS010000000)

To characterize the virulence-associated repertoire of the *Vibrio cholerae* isolate (JCASQS010000000), the polished genome assembly was screened against the Virulence Factor Database (VFDB). The identified genes were classified according to their predicted functional roles and genomic locations. The complete set of putative virulence-associated determinants detected in the isolate is presented in Table 4.

VFDB screening identified 24 virulence-associated determinants. These loci were mainly associated with quorum sensing, Type VI secretion system components and effectors, RTX toxin-associated functions, and cytolysin production. The VFDB hits showed high overall similarity to reference sequences, with coverage values ranging from 91.70% to 100.00% and sequence identity values ranging from 91.01% to 99.78%.

Complementary analysis of major *V. cholerae* virulence-associated gene clusters revealed marked differences in their completeness. The toxin-coregulated pilus (TCP) cluster was completely absent, with none of the 14 investigated TCP-associated genes (*tcpA*, *tcpB*, *tcpC*, *tcpD*, *tcpE*, *tcpF*, *tcpH*, *tcpI*, *tcpJ*, *tcpN*, *tcpQ*, *tcpR*, *tcpS*, and *tcpT*) detected in the genome. In contrast, the Lux quorum-sensing operon was complete, with all five investigated genes (*luxO*, *luxP*, *luxQ*, *luxS*, and *luxU*) present as complete and non-disrupted sequences, exhibiting nucleotide identities ranging from 94.76% to 99.42%.

The RTX toxin-associated locus showed an incomplete configuration. The *rtxB*, *rtxC*, and *rtxD* genes were present as complete and non-disrupted sequences, whereas *rtxA* was represented by two disrupted fragments. Similarly, the mannose-sensitive haemagglutinin (MSHA) pilus cluster was incomplete. Ten of the 13 genes (*mshB*, *mshD*, *mshE*, *mshF*, *mshG*, *mshH*, *mshI*, *mshJ*, *mshK*, and *mshM*) were detected as complete sequences, whereas *mshA*, *mshC*, and *mshN* were classified as incomplete.

To further assess the predicted pathogenic potential of the *V. cholerae* isolate, the genome assembly was analyzed using PathogenFinder2 v0.6.0. The software estimates the probability that a bacterial genome shares genomic characteristics associated with human pathogenicity through an ensemble of four neural network models and a classification threshold of 0.5.

All four neural network models produced high pathogenicity probability scores (NN1, 0.9316; NN2, 0.9736; NN3, 0.9736; and NN4, 0.9761), with a mean probability score of 0.9637 ± 0.0186. As this value substantially exceeded the predefined classification threshold, the isolate was consistently classified as a potential human pathogen across all models.

Overall, the isolate exhibited a substantial repertoire of virulence-associated determinants, including a Type VI secretion system, *hlyA*, a complete *lux* quorum-sensing operon, and components of the RTX toxin and MSHA pilus systems. However, the complete absence of the TCP cluster and the disrupted configuration of *rtxA*, together with the incomplete MSHA locus, indicate that several major virulence-associated systems were absent or structurally incomplete. The high PathogenFinder2 probability therefore supports a genome-wide association with human pathogenicity but should be interpreted in the context of this specific virulence-gene profile rather than as direct evidence of a fully virulent or toxigenic phenotype.

#### 3.4.2. *Vibrio parahaemolyticus* Isolate (JCASQT010000000)

The polished genome assembly of the *Vibrio parahaemolyticus* isolate (JCASQT010000000) was screened against the Virulence Factor Database to characterize its putative virulence-associated gene repertoire. The detected determinants were classified according to their predicted functional roles and genomic locations. The complete virulence-associated profile of the isolate is presented in Table 5.

In silico screening against VFDB revealed multiple virulence-associated determinants in the *V. parahaemolyticus* assembly. On the larger chromosome, the analysis identified VPA0450, encoding a T3SS1-associated effector, and *tlh*, encoding thermolabile hemolysin. Most virulence factor genes were located on the smaller chromosome, including genes associated with motility and chemotaxis (*fli*G, *che*Y, *che*W, *flg*B), adherence (*vpad*F, *mam*7), and an extensive Type III secretion system 1 locus comprising regulatory, structural, chaperone, translocator and effector genes. Overall, VFDB hits showed high gene coverage, ranging from 94.27% to 100%, with sequence identity values between 80.25% and 100%. Most T3SS1-associated genes displayed complete coverage and high sequence identity, supporting the presence of a well conserved T3SS1 associated region in this isolate.

To evaluate the predicted pathogenic potential of the *V. parahaemolyticus* isolate, the genome assembly was analyzed using PathogenFinder2 v.0.6.0. The computational analysis yielded high pathogenic probability scores across the neural network modules, although with some variation among the models (NN1: 0.9146; NN2: 0.6597; NN3: 0.9683; NN4: 0.9170), resulting in a mean output score of 0.8649 (±0.1204). While one of the modules (NN2) presented a more moderate score, the overall mean is substantially higher than the 0.5 threshold, moving the sample well beyond the model’s decision boundary.

Accordingly, the genome of this *V. parahaemolyticus* isolate was predicted to possess human pathogenic capacity. The presence of genome-wide sequence patterns and virulence-associated determinants previously described in pathogenic bacteria suggests that the isolate may possess traits associated with pathogenic potential. However, these in silico findings should not be interpreted as direct evidence of its ability to cause human infection.

### 3.5. Mobile Genetic Elements: Plasmids and Prophages

#### 3.5.1. *Vibrio cholerae* Isolate (JCASQS010000000)

Prophage prediction identified two putative prophage-associated regions in the *Vibrio cholerae* genome, of which one was classified as intact and the other as incomplete. The incomplete region was located between positions 599,884 and 621,358 bp of contig 1, spanning approximately 21.4 kb, and obtained a prophage completeness score of 30. This region contained 11 predicted proteins, including 10 proteins with matches to phage-associated sequences and one hypothetical protein, collectively representing 100% of the encoded proteins. One tRNA and a putative attachment site were also identified within this region. Phage-related annotations included integrase- and tail-associated proteins. The most frequent phage similarity was observed with *Escherichia* phage 500465-1 (7/11 proteins; 63.6%), followed by lower numbers of matches to Enterobacteria phages P4 and N15 and several other phages. The region exhibited a GC content of 44.70%, which was lower than the overall genome GC content of 47.55%.

The second prophage-associated region was located between positions 1,238,354 and 1,247,855 bp of contig 1 and spanned approximately 9.5 kb. This region obtained a completeness score of 109 and was therefore classified as an intact prophage. It contained 19 predicted proteins, comprising 12 proteins with significant phage-associated matches and seven hypothetical proteins, together accounting for 100% of the proteins encoded within the region. A capsid-associated protein was identified, whereas no tRNA or recognizable attachment site was detected. The most common matches corresponded to *Vibrio* phages KSF-1φ and VCY-φ, each represented by eight matching proteins, followed by VGJφ, VEJφ and VfO3K6, each represented by five matches. Overall, the region showed similarities predominantly to previously characterized filamentous *Vibrio* phages. Its GC content was 44.65%, also lower than that of the complete genome assembly.

No plasmid replicons were detected by the *in silico* analyses.

#### 3.5.2. *Vibrio parahaemolyticus* Isolate (JCASQT010000000)

Prophage prediction identified seven putative prophage-associated regions distributed across the *Vibrio parahaemolyticus* genome assembly. Collectively, these regions encompassed approximately 150.1 kb, corresponding to 2.85% of the 5.26 Mb genome assembly. Based on the prediction scores, two regions (Regions 1 and 6) were classified as intact and five (Regions 2–5 and 7) as incomplete, while no questionable regions were identified. Within the predicted boundaries, the annotated proteins consisted exclusively of phage-associated and hypothetical proteins, with no bacterial proteins identified.

Among the two regions classified as intact, Region 1 was the largest and most structurally complex. Located at positions 955,210–1,009,445 bp of contig 4, it spanned 54.2 kb and received a completeness score of 136. The region encoded 63 predicted proteins, including 23 with phage-associated database hits and 40 hypothetical proteins. Several structural and mobility-associated components were identified, including head-, capsid-, portal-, and tail-associated proteins, together with a transposase. The predominant database similarity was to *Vibrio* phage VCYφ, with ten protein matches, although additional similarities to other *Vibrio*-associated phages were also detected. No recognizable attachment site or tRNA gene was identified, and the region exhibited a GC content of 42.55%. Region 6 was likewise classified as intact and received a completeness score of 136 but was considerably smaller, spanning 7.2 kb at positions 2,328,032–2,335,241 bp of contig 1. It encoded 11 predicted proteins, ten of which showed phage-associated matches and one of which was hypothetical. All ten phage-associated proteins showed similarity to *Vibrio* phage VCYφ, although a capsid-associated protein was the only specifically identifiable structural component. No attachment site or tRNA gene was detected, and the GC content was 42.75%.

The remaining five regions were classified as incomplete and varied in size from 10.1 to 33.7 kb, with completeness scores ranging from 20 to 50. Region 2, located at positions 172,294–186,897 bp of contig 1, spanned 14.6 kb and received the lowest completeness score of 20. It encoded 15 predicted proteins, including eight phage-associated matches and seven hypothetical proteins. A baseplate-associated protein was the only recognizable phage structural feature. Although no attachment site was detected, the region contained one tRNA gene. Four proteins showed similarity to *Klebsiella* phage ST147-VIM1φ7.1, representing the predominant database match, and the region had a GC content of 44.82%.

Region 3 extended from positions 1,873,207 to 1,883,338 bp of contig 1, spanning 10.1 kb, and received a completeness score of 30. It encoded 15 predicted proteins, including 11 with phage-associated matches and four hypothetical proteins. Integrase- and portal-associated proteins were identified, together with a predicted attachment site, whereas no tRNA gene was detected. Four proteins showed similarity to *Vibrio* phage 12A4, which represented the predominant database match. The GC content of this region was 41.41%.

Region 4 was the largest of the incomplete regions, spanning 33.7 kb at positions 1,892,966–1,926,706 bp of contig 1 and receiving a completeness score of 50. It encoded 23 predicted proteins, including 20 with phage-associated hits and three hypothetical proteins. Terminase-, portal-, and capsid-associated proteins were identified, while no attachment site or tRNA gene was detected. Seventeen proteins showed similarity to *Vibrio* phage 12A4, making it the predominant database match. The region exhibited a GC content of 44.65%.

Region 5 was located at positions 2,085,831–2,099,847 bp of contig 1 and spanned 14.0 kb. With a completeness score of 30, it encoded 14 predicted proteins, equally divided between proteins with phage-associated hits and hypothetical proteins. Integrase- and transposase-associated proteins were detected, together with a predicted attachment site, whereas no tRNA gene was present. Its proteins showed heterogeneous similarities to several reference phages, with no single predominant match. The GC content of Region 5 was 43.42%.

Finally, Region 7 encompassed positions 2,586,019–2,602,342 bp of contig 1, spanning 16.3 kb and receiving a completeness score of 40. It encoded 19 predicted proteins, including 13 with phage-associated matches and six hypothetical proteins. Tail- and integrase-associated proteins were identified, together with a predicted attachment site, although no tRNA gene was detected. As observed for Region 5, the detected proteins showed heterogeneous similarities to phages associated with different bacterial hosts, without a clearly predominant database match. Region 7 exhibited the lowest GC content among all predicted prophage-associated regions, at 40.76%.

Overall, the seven predicted regions exhibited GC contents ranging from 40.76% to 44.82%, consistently below the genome-wide GC content of 45.19%. This compositional difference, together with the occurrence of integrases, attachment sites, mobility-associated proteins, and structural phage components across several regions, is compatible with the presence of horizontally acquired phage-derived elements. However, these classifications are based on computational predictions of prophage completeness and do not demonstrate prophage inducibility or replication competence.

### 3.6. Phylogenomic Analysis

#### 3.6.1. *Vibrio cholerae*

**Figure 3:**
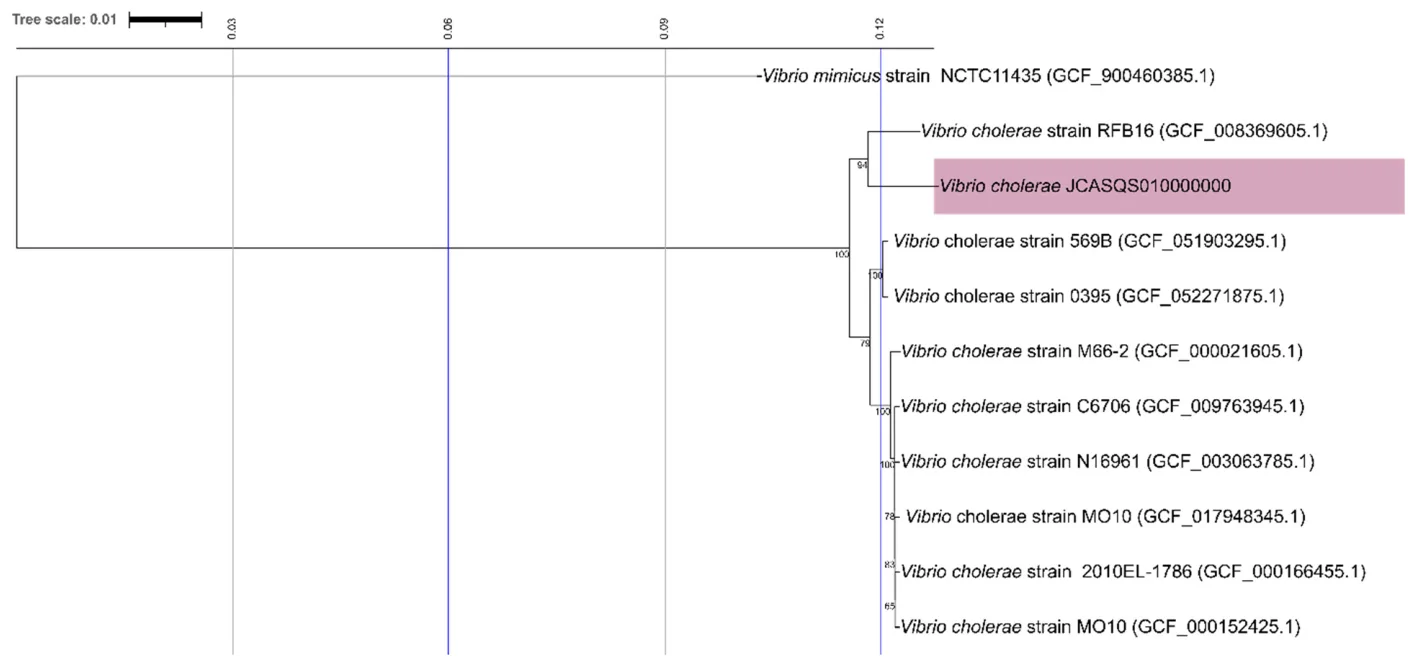
Maximum-likelihood phylogenomic analysis of the *Vibrio cholerae* isolate JCASQS010000000. The phylogenetic tree was reconstructed from a core-genome alignment using IQ-TREE2 under the TIM2+F+I+R4 nucleotide substitution model with 1000 ultrafast bootstrap replicates. The study isolate (JCASQS010000000) is highlighted in pink. *Vibrio mimicus* strain NCTC11435 (GCF_900460385.1) was included as an outgroup. Branch lengths represent the estimated number of nucleotide substitutions per site, as indicated by the scale bar. Bootstrap support values are shown at the corresponding internal nodes.

Maximum-likelihood phylogenomic analysis positioned *Vibrio cholerae* isolate JCASQS010000000 as the sister taxon of *V. cholerae* strain RFB16 (GCF_008369605.1), with strong bootstrap support (94%; Figure 3). The clade formed by these two genomes was separated from all other *V. cholerae* reference genomes included in the analysis by a fully supported internal node (100% bootstrap support). The remaining strains formed a distinct cluster comprising classical biotype reference genomes, including 569B and O395, together with El Tor- and O139-associated strains such as N16961, C6706, MO10, M66-2, and 2010EL-1786. The phylogeny was rooted using *Vibrio mimicus* strain NCTC11435 (GCF_900460385.1) as the outgroup. Collectively, these results identify RFB16 as the closest phylogenetic relative of JCASQS010000000 among the genomes included in the analysis and place the study isolate within a lineage distinct from the principal pandemic reference lineages represented in the dataset.

#### 3.6.2. Phylogenomic Analysis of *Vibrio parahaemolyticus* Isolate

**Figure 4:**
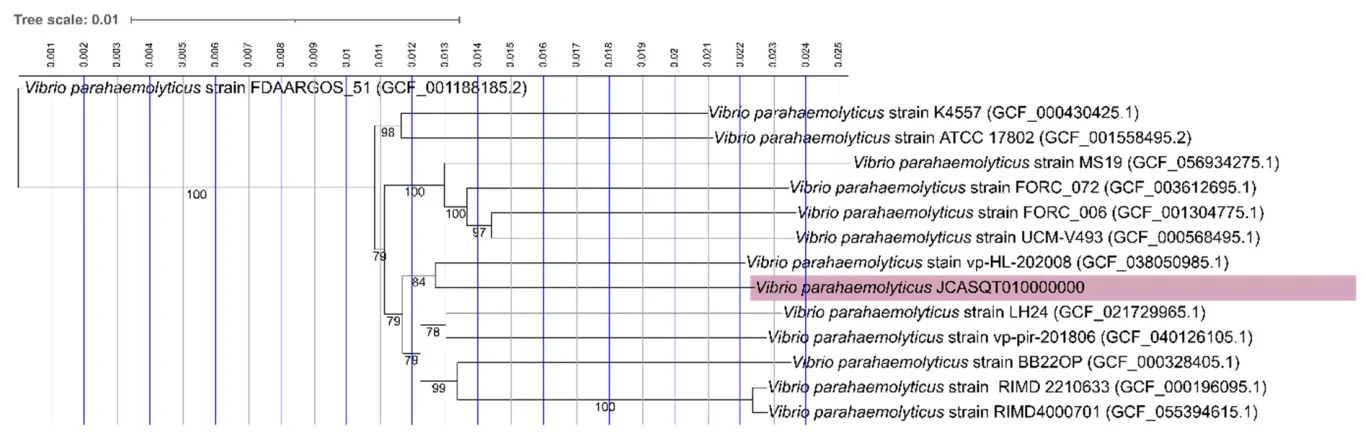
Maximum-likelihood phylogenomic analysis of the *Vibrio parahaemolyticus* isolate JCASQT010000000. The phylogenetic tree was reconstructed from a core-genome alignment using IQ-TREE2 under the GTR+F+I+R3 nucleotide substitution model with 1000 ultrafast bootstrap replicates. The study isolate (JCASQT010000000) is highlighted in pink. The phylogeny was rooted using *V. parahaemolyticus* strain FDAARGOS_51 (GCF_001188185.2). Branch lengths represent the estimated number of nucleotide substitutions per site, as indicated by the scale bar, and bootstrap support values are shown at the corresponding internal nodes.

Maximum-likelihood phylogenomic analysis positioned the *Vibrio parahaemolyticus* isolate JCASQT010000000 among the reference genomes included in the dataset (Figure 4). The study isolate formed a sister relationship with *V. parahaemolyticus* strain vp-HL-202008 (GCF_038050985.1), supported by a bootstrap value of 84%, identifying this strain as its closest phylogenetic relative among the genomes examined. This lineage was distinct from the cluster comprising the pandemic reference strains RIMD 2210633 (GCF_000196095.1) and RIMD4000701 (GCF_055394615.1), which grouped together with 100% bootstrap support. For visualization purposes, the phylogeny was displayed using *V. parahaemolyticus* strain FDAARGOS_51 (GCF_001188185.2) as an internal reference root.

Overall, the topology places JCASQT010000000 within a lineage represented by strain vp-HL-202008 and separated from the RIMD 2210633/RIMD4000701 lineage within the set of genomes analyzed.

## 4. Discussion

This study provides a whole-genome-based characterization of *Vibrio cholerae* and *Vibrio parahaemolyticus* isolates recovered from commercially depurated mussels and clams purchased at retail in Portugal. Although the analysis was limited to two isolates and therefore cannot provide estimates of prevalence or exposure, their recovery from products considered suitable for commercial distribution is relevant from a food-safety perspective. Conventional monitoring of live bivalve mollusks is largely centered on fecal indicators and enteric pathogens and does not necessarily capture autochthonous marine bacteria such as *Vibrio* spp. [5]. Moreover, experimental and field studies have demonstrated that members of the natural *Vibrio* community may persist in bivalve tissues during depuration, with clearance efficiency depending on bacterial, host, and process-related factors [50,51]. Nevertheless, because bacterial concentrations were not determined and matched pre- and post-depuration samples were unavailable, the present findings cannot be interpreted as evidence of depuration failure or used to quantify the effectiveness of the commercial process.

WGS was essential for resolving the taxonomic identity of both isolates after inconclusive automated identification. ANI analysis supported their assignment to *V. cholerae* and *V. parahaemolyticus*, while the chromosomes were consistent with the characteristic bipartite genome architecture of the genus [52,53,54]. The high contiguity of the assemblies additionally allowed the genomic context of resistance- and virulence-associated determinants to be investigated and facilitated the identification of prophage-associated regions. These findings emphasize the value of genome-based identification and characterization of environmental *Vibrio* isolates, which may be underrepresented in databases supporting biochemical identification systems developed predominantly from clinically encountered organisms.

The scientific contribution of the present study lies in the detailed characterization of two archived seafood-associated *Vibrio* isolates exhibiting specific genomic features that could not be resolved through conventional phenotypic identification. Although neither isolate displayed the major genomic determinants associated with classical pandemic lineages, their characterization revealed informative differences in antigen-associated loci, antimicrobial resistance profiles, and virulence-associated genomic repertoires. In particular, the mussel-derived *V. cholerae* isolate exhibited an unresolved O1/O139 serogroup assignment despite the detection of a complete *wbfZ*-like sequence, together with an extensive Type VI secretion system repertoire and an elevated meropenem MIC without an identifiable acquired carbapenemase. Similarly, the clam-derived *V. parahaemolyticus* ST98 isolate carried a divergent KL55-like K-antigen locus, highlighting genomic variation that could not be adequately characterized through routine identification or MLST alone. The characterization of resistance-associated determinants, prophage regions, and phylogenomic relationships further provides strain-level genomic information beyond conventional microbiological approaches [55]. Importantly, the availability of both genome assemblies in public databases enables their inclusion in future comparative investigations. Accordingly, the value of these findings lies in documenting the specific genomic characteristics of two archived isolates recovered from commercially depurated bivalves, rather than establishing the prevalence, population diversity, or pathogenic potential of *Vibrio* spp. in seafood [55].

The mussel-derived *V. cholerae* isolate displayed a genomic profile clearly distinct from that of classical toxigenic pandemic strains included in the analysis. The absence of the O1-associated *rfbV* marker did not support an O1 assignment, whereas the complete but non-exact *wbfZ*-like sequence, with 96.42% nucleotide identity, was insufficient to establish an O139 genotype. Similar high-identity *wbfZ*-like matches have previously generated ambiguous or potentially misleading O139 predictions, underlining the need to examine the entire O-antigen biosynthesis locus and, where possible, confirm the result serologically. The isolate should therefore remain unassigned with respect to O1/O139 status until complete locus-level characterization is performed [56].

The absence of *ctxA*, *ctxB*, the toxin-coregulated pilus cluster, *ace*, and *zot* indicates that this isolate lacks the major genomic determinants associated with the canonical toxigenic cholera phenotype. This finding is also consistent with its separation from the classical and seventh-pandemic reference genomes included in the phylogenomic analysis [53,57]. Nevertheless, the absence of CTXφ and TCP should not be equated with biological innocuity. This interpretation is consistent with observations from European coastal non-O1/non-O139 *V. cholerae*, in which environmental isolates lacking the classical epidemic cholera profile nevertheless carried diverse toxin- and secretion-system-associated determinants and, in functional assays, displayed phenotypes including hemolysis, biofilm formation, and, in a subset of strains, survival in human serum [58]. The genome carried several determinants potentially involved in environmental competition and opportunistic host interaction, including a substantial Type VI secretion system repertoire, *hcp*- and *vgrG*-associated components, *hlyA*, *chxA*, *makA*, *hapA* and quorum-sensing determinants. In *V. cholerae*, LuxS-mediated AI-2 quorum sensing contributes to cell-density-dependent regulation through the LuxPQ/HapR pathway, influencing biofilm formation and the expression of major virulence determinants, including cholera toxin and the toxin-coregulated pilus [47,48].

Environmental and non-O1/non-O139 *V. cholerae* populations frequently harbor T6SS, hemolysin, and RTX-associated loci despite lacking the principal cholera toxin machinery [53,57,59]. T6SS activity may enhance competitive fitness in polymicrobial aquatic communities, whereas cytolysin and other toxin-associated proteins can contribute to membrane damage or cytotoxicity under appropriate conditions [57]. However, the incomplete *rtxA* sequence identified here argues against assuming the presence of a fully functional RTX toxin system, and the detection of these determinants alone does not demonstrate their expression, secretion, or contribution to disease.

Recent population-genomic analysis of non-O1/non-O139 *V. cholerae* further highlights the heterogeneous distribution of virulence-associated determinants across environmental and clinical populations [60]. Notably, environmental isolates recovered from food sources exhibited virulence-gene profiles more similar to those of clinical isolates than did isolates from other environmental sources, and determinants such as *hlyA*, *toxR*, *hapA*, *chxA*, and secretion-system-associated genes were distributed across both groups. These observations reinforce the view that the absence of the canonical cholera virulence machinery does not necessarily imply the absence of other traits potentially relevant to host interaction, while also emphasizing that gene content alone is insufficient to predict pathogenicity [60].

Phylogenomically, JCASQS010000000 was positioned as the sister taxon of *V. cholerae* RFB16, an environmental strain originally recovered from a freshwater lake in Pennsylvania [61]. This relationship is biologically coherent with the non-pandemic genomic profile of the study isolate and with the aquatic environmental diversity that characterizes the species. The JCASQS010000000–RFB16 grouping received 94% ultrafast-bootstrap support and was separated from the pandemic reference cluster by a fully supported internal branch. The relationship should nevertheless be interpreted within the limits of the reference dataset, with RFB16 representing the closest relative among the genomes examined. The present tree does not capture the full diversity of environmental *V. cholerae* and therefore cannot establish the geographical distribution, population frequency or epidemiological status of this lineage.

The clam-derived *V. parahaemolyticus* isolate was unambiguously assigned to ST98 through exact and complete matches at all seven MLST loci [45]. Although this provides a reproducible nomenclatural designation, MLST assignment alone does not define virulence, ecological origin or epidemiological relatedness, particularly in a highly diverse and recombinogenic species such as *V. parahaemolyticus*. O-antigen locus typing supported OL4, whereas the K-antigen locus was most similar to KL55. The absence of KL55_04 and the additional presence of KL69_04- and KL4_06-like genes indicate that the region differs from the canonical KL55 reference and may represent a divergent or mosaic capsule locus. The most defensible designation is therefore OL4:KL55-like rather than a definitive O4:K55 serotype. Such variation is biologically plausible because the *V. parahaemolyticus* O- and K-antigen regions are evolutionarily dynamic and may be remodeled through recombination, horizontal gene transfer, and gene-content variation.

The virulence-associated repertoire of JCASQT010000000 was dominated by a broad T3SS1-associated gene set, including regulatory, structural, chaperone, translocator, and effector components. Additional determinants included *tlh*, VPA0450, *vpadF*, *mam7*, and genes associated with motility and chemotaxis. T3SS1 contributes to host–cell manipulation and cytotoxicity, whereas adhesins and motility-associated systems may support attachment, colonization, and environmental persistence. Nevertheless, T3SS1 and *tlh* are widely distributed within *V. parahaemolyticus* and are not sufficient to distinguish clinically virulent strains from environmental populations. In contrast, the absence of *tdh* and *trh*, which remain important epidemiological markers associated with many human gastroenteritis isolates, argues against classifying JCASQT010000000 as a conventional enteropathogenic or pandemic strain [62]. Its profile is therefore more appropriately interpreted as a virulence-associated genomic background with potential for host interaction rather than direct evidence of human pathogenicity.

The phylogenomic placement of JCASQT010000000 provides additional context. The isolate grouped most closely with strain vp-HL-202008, with 84% ultrafast-bootstrap support, and was separated from the RIMD 2210633/RIMD4000701 pandemic reference lineage included in the analysis. Strain vp-HL-202008 belongs to a group of *V. parahaemolyticus* isolates recovered from diseased shrimp during highly lethal *Vibrio* disease, also referred to as translucent post-larvae disease, in China [63]. However, the moderate support for this relationship and the proximity of the two genomes in a core-genome tree do not imply conservation of accessory virulence determinants. The severe shrimp phenotype of the vp-HL strains has been linked to plasmid-encoded toxin systems, and no equivalent plasmid-mediated toxin repertoire was demonstrated for JCASQT010000000 in the present study [63]. Consequently, the observed clustering does not support describing the study isolate as hypervirulent, shrimp pathogenic, or ecologically equivalent to vp-HL-202008.

PathogenFinder produced scores above the predefined classification threshold for both genomes. These outputs indicate that the isolates contain genome-wide sequence patterns associated with human pathogenicity according to the model but do not measure virulence directly or establish that either isolate can cause human infection. This distinction is particularly important for environmental vibrios, in which secretion systems, hemolysins, adhesins, and stress-response determinants may contribute to both aquatic fitness and opportunistic host interaction [57]. The PathogenFinder predictions should therefore be considered complementary to locus-specific analysis and interpreted in the context of the complete genomic profile, including the absence of major pathotype-defining determinants [64]. Functional assessment of hemolysis, adhesion, cytotoxicity, colonization and toxin expression would be required to determine the biological relevance of the predicted pathogenicity scores [58].

The antimicrobial susceptibility findings require equally conservative interpretation. Under the current EUCAST v.16.1 *Vibrio* spp. breakpoints, a meropenem MIC of 4 mg/L supports classification of the isolate as resistant, as it exceeds the resistance breakpoint of 0.5 mg/L. In contrast, the censored MIC results reported as cefotaxime (≤1 mg/L), piperacillin–tazobactam (≤8 mg/L) and trimethoprim–sulfamethoxazole (≤2/38 mg/L) do not allow determination of whether the actual MIC lies above or below the corresponding breakpoint. Genotypic resistance determinants should be interpreted as complementary mechanistic evidence rather than as a substitute for phenotypic susceptibility testing. This is particularly relevant for colistin, for which the presence or absence of *mcr*-like genes alone is insufficient to infer resistance or susceptibility. In *Vibrio* spp., polymyxin resistance may also involve intrinsic chromosomal mechanisms, including modifications of lipid A and lipopolysaccharide, which may not be adequately captured by conventional ARG databases. Thus, WGS can provide supportive evidence of potential resistance mechanisms but should not replace phenotypic MIC determination. For tobramycin, the detection of aminoglycoside resistance determinants may support genotype–phenotype concordance when the predicted resistance spectrum of the identified gene includes this antimicrobial, although categorical interpretation remains limited in the absence of species-specific clinical breakpoints. Moreover, EUCAST does not currently provide *Vibrio*-specific clinical breakpoints for colistin or tobramycin, and these results should therefore be presented as MICs without categorical S/R interpretation unless another explicitly justified interpretive criterion is applied [27]. Confirmation using a reference MIC method would strengthen the interpretation of uncertain or clinically relevant findings, particularly the meropenem and colistin results.

The genomic resistance profiles were predominantly composed of chromosomal resistance-associated determinants. The *V. cholerae* isolate lacked an identifiable acquired carbapenemase but carried the *vex* RND-efflux system, *vcmA*, *almG*, and the global regulator *crp*. These loci form part of the intrinsic adaptive repertoire of *V. cholerae* and should not be interpreted as horizontally acquired ARGs or as direct explanations for the elevated meropenem MIC [61]. Regulatory changes, altered membrane permeability, porin variation, or modifications affecting antimicrobial targets may contribute to the observed phenotype and would require targeted investigation. In the *V. parahaemolyticus* genome, the detection of a chromosomal *bla*CARB-like determinant despite a low ampicillin MIC illustrates the limited predictive value of gene presence without information on allele functionality and expression. The potential contribution of *tet*(35) and the moderately similar *tet*(34)-like sequence could not be evaluated phenotypically because tetracyclines were not included in the susceptibility panel. Overall, these findings reinforce the need to integrate standardized phenotypic testing with allele-level genomic analysis rather than treating database hits as independent evidence of resistance.

The prophage-associated regions detected in both genomes further illustrate the contribution of mobile DNA to *Vibrio* genome plasticity [62]. The *V. cholerae* genome contained two predicted regions, including a putatively intact element with similarities to filamentous *Vibrio* phages, whereas seven regions were detected in JCASQT010000000. The lower GC content of several regions relative to their host genomes, together with the presence of integrases, attachment sites, and phage structural proteins, is compatible with horizontal acquisition [62]. Nevertheless, similarity to several reference phages is more indicative of mosaic ancestry than of identity with a particular named phage. Likewise, computational classification of prophage completeness does not demonstrate inducibility, particle production, or infectivity. This caveat is particularly relevant to the small 7.2 kb *V. parahaemolyticus* region, whose limited size and structural repertoire warrant cautious interpretation as an independently functional prophage.

From a One Health perspective, bivalves occupy an important interface between coastal microbial communities, food production, and human exposure. The present study does not demonstrate an immediate consumer-health risk, nor does it indicate that the isolates were present at infectious concentrations. It does, however, demonstrate the recovery of two distinct Vibrio species from commercially depurated retail bivalves, each exhibiting isolate-specific genomic characteristics that are not assessed through conventional fecal-indicator-based monitoring. This observation is relevant in the context of changing coastal environmental conditions and the recognized importance of seafood-associated *Vibrio* hazards in Europe [63]. Targeted, harmonized surveillance incorporating quantitative detection, isolate recovery and genomic characterization could complement existing monitoring frameworks and provide data relevant to future risk assessment.

Several limitations should be considered. The study examined only two selected isolates obtained following enrichment, precluding estimates of prevalence, bacterial abundance, or population diversity. The lack of paired samples before and after depuration prevented direct evaluation of bacterial removal. Antimicrobial susceptibility testing was constrained by the restricted concentration ranges and interpretive limitations of the automated MicroScan panel, and selected MIC findings would benefit from confirmation using reference methods. In addition, the lack of species-specific clinical breakpoints for several antimicrobial agents limits categorical interpretation of susceptibility results in *Vibrio* spp. The pathogenicity assessments were exclusively computational and were not supported by gene-expression or functional assays. In addition, the exact O-antigen locus of the *V. cholerae* isolate, the structure and phenotypic expression of the KL55-like locus, the origin of the additional 8.2 kb *V. parahaemolyticus* contig, and the functionality of the predicted prophages remain unresolved. A further limitation of this study is the historical nature of the isolates, which were recovered from bivalves sampled in 2016. Therefore, the present findings should not be interpreted as representative of the current epidemiological situation of *Vibrio* spp. in bivalves marketed in Portugal. Nevertheless, the genomic characterization of these archived isolates provides retrospective, isolate-specific information on antimicrobial resistance-associated determinants, virulence-associated features, and phylogenomic relationships in two Vibrio strains recovered from commercially marketed bivalves in 2016. These genomic data may serve as reference points for future comparative studies, although they cannot establish broader population diversity, circulation patterns, or temporal trends. Finally, the phylogenomic analyses incorporated a limited reference genome set and therefore provide comparative placement rather than a comprehensive population genomic assessment of either species.

## 5. Conclusions

WGS enabled the identification and detailed genomic characterization of *Vibrio cholerae* and *Vibrio parahaemolyticus* isolates recovered from commercially depurated Portuguese bivalves. The *V. cholerae* isolate lacked CTXφ-, the TCP cluster, and other major determinants associated with toxigenic cholera and was phylogenomically associated with the environmental freshwater strain RFB16 rather than with the pandemic reference lineages represented in the analysis. Nevertheless, it carried T6SS-, hemolysin-, and toxin-associated determinants that may contribute to environmental fitness and, under appropriate conditions, opportunistic host interaction. Its O1/O139 status remained unresolved and requires full O-antigen locus characterization.

The *V. parahaemolyticus* isolate was assigned to ST98 and OL4 and carried a divergent KL55-like K-antigen locus. Its genome encoded an extensive T3SS1-associated repertoire, together with adhesion, motility, and hemolysin-associated determinants, but lacked the major enteropathogenic markers *tdh* and *trh*. Phylogenomically, the isolate was separated from the RIMD 2210633/RIMD 4000701 pandemic reference lineage included in the analysis. Although it grouped most closely with the shrimp-associated strain vp-HL-202008, the moderate branch support and absence of demonstrated equivalence in plasmid-encoded toxin content do not support an inference of shared hypervirulence or ecological behavior.

Collectively, the findings reveal two *Vibrio* isolates containing distinct combinations of intrinsic resistance-associated determinants, virulence-associated systems, and prophage-derived regions. Their recovery does not establish depuration failure or an immediate consumer health risk but demonstrates that fecal indicator-based compliance does not exclude the presence of autochthonous *Vibrio* spp. in retail bivalves. Broader longitudinal surveillance combining quantitative microbiology, reference antimicrobial susceptibility testing, expanded phylogenomic analysis, complete antigen-locus characterization and functional assessment of virulence-associated traits will be important to determine the distribution and public-health relevance of related *Vibrio* populations in Portuguese seafood, particularly in the context of ongoing changes in coastal environmental conditions and ocean warming.

## Figures and Tables

**Table 1:** Antimicrobial susceptibility testing results for the mussel-derived *Vibrio cholerae* isolate (JCASQS010000000) and the clam-derived *Vibrio parahaemolyticus* isolate (JCASQT010000000), recovered from *Mytilus* spp. and *Spisula solida*, respectively. MIC, minimum inhibitory concentration; R, resistant; S, susceptible; NI, not interpretable; NA, not applicable.

Antimicrobial Agent	*V. cholerae* MIC (mg/L)	Interpretation	*V. parahaemolyticus* MIC (mg/L)	Interpretation
Ampicillin	≤4.00	NA	≤4.00	NA
Cefepime	≤1.00	NA	≤1.00	NA
Cefotaxime	≤1.00	NI	≤1.00	NI
Ceftazidime–clavulanate	≤0.25	NA	≤0.25	NA
Ertapenem	≤0.12	NA	≤0.12	NA
Meropenem	4	R	≤0.12	S
Nalidixic acid	≤16.00	NA	≤16.00	NA
Norfloxacin	≤0.50	NA	≤0.50	NA
Amikacin	≤8.00	NA	≤8.00	NA
Gentamicin	≤2.00	NA	≤2.00	NA
Tobramycin	≤2.00	NA	4	NA
Trimethoprim–sulfamethoxazole	≤2/38	NI	≤2/38	NI
Aztreonam	≤1.00	NA	≤1.00	NA
Ceftazidime	≤1.00	S	≤1.00	S
Cefoxitin	≤8.00	NA	≤8.00	NA
Cefuroxime	≤8.00	NA	≤8.00	NA
Colistin	4	NA	≤2.00	NA
Fosfomycin	32	NA	≤8.00	NA
Nitrofurantoin	≤64.00	NA	≤64.00	NA
Cefotaxime–clavulanate	≤0.25	NA	≤0.25	NA
Piperacillin–tazobactam	≤8.00	NI	≤8.00	NI

NA, no EUCAST v16.1 clinical breakpoint available for *Vibrio* spp.; NI, not interpretable because the MIC range provided by the commercial panel encompasses the applicable EUCAST breakpoint.

**Table 2:** Antimicrobial resistance-associated determinants identified in the genome of the *Vibrio cholerae* isolate (JCASQS010000000). Detected determinants were grouped according to their functional category, genomic location, and predicted resistance mechanism or function.

Functional Category	Resistance-Associated Determinants	Genomic Location	Predicted Resistance Mechanism/Function
Polymyxins/Cationic antimicrobial peptides	*alm*G	Chromosome 1′	Lipid A modification, reducing susceptibility to cationic antimicrobial peptides such as polymyxins.
Multidrug resistance (MDR)/Drug and biocide efflux pumps	*vex*A, *vex*B, *vex*C, *vex*D, *vex*E, *vex*F, *vex*H, *vex*K	Chromosome 1′	Active expulsion of antimicrobial compounds and biocides through RND efflux pumps.
Multidrug resistance (MDR)/MATE efflux pump	*vcm*A	Chromosome 1′	Active extrusion of structurally diverse antimicrobial compounds through a MATE-family efflux pump.
Efflux-associated resistance regulators	*crp*	Chromosome 1′	Global regulatory control of multidrug efflux pump expression, potentially influencing resistance to fluoroquinolones, macrolides and β-lactams.

**Table 3:** Antimicrobial resistance-associated determinants identified in the genome of the *Vibrio parahaemolyticus* isolate (JCASQT010000000). Detected determinants are grouped according to functional category, genomic location, and predicted resistance mechanism.

Functional Category	Resistance-Associated Determinants	Genomic Location	Predicted Resistance Mechanism/Function
β-lactams/Penicillins	*bla*CARB-like	Chromosome 1′	Class A β-lactamase-mediated hydrolysis of β-lactam antibiotics, mainly associated with resistance to penicillins such as ampicillin, amoxicillin and piperacillin.
Tetracyclines	*tet*(35)	Chromosome 2′	Active efflux of tetracycline-class antimicrobials through a membrane-associated transporter, reducing intracellular drug accumulation.
Tetracyclines	*tet*(34)-like	Chromosome 2′	Putative tetracycline resistance determinant associated with reduced susceptibility to tetracyclines; conservatively reported as *tet*(34)*-like* due to moderate sequence identity.

**Table 4:** Virulence-associated determinants identified in the genome of the *Vibrio cholerae* isolate (JCASQS010000000). Detected determinants are grouped according to functional category, genomic location, and predicted biological role.

Functional Category	Virulence-Associated Determinants	Genomic Location	Predicted Biological Role
Quorum sensing/AI-2 signaling	*luxS*	chromosome 1	AI-2 autoinducer synthesis and quorum-sensing-mediated regulation of virulence-associated phenotypes, including the ToxR/ToxT virulence cascade, biofilm formation, motility, and host-associated adaptation [47,48,49].
Type VI secretion system: structural and regulatory components	*vasA*, *vasB*, *vasC*, *vasD*, *vasE*, *vasF*, *clpB/vasG*, *vasH*, *vasI*, *vasJ*, *icmF/vasK*, *vasL*, *VCA0109*, *VCA0122*, *vipA/mglA*, *vipB/mglB*	chromosome 2	Assembly and regulation of the Type VI secretion system, involved in interbacterial competition, host interaction, and environmental fitness.
Type VI secretion system: effectors/substrates	*hcp-2*, *vgrG-2*, *vgrG-3*	chromosomes 1 and 2	Secreted T6SS-associated proteins involved in effector delivery, bacterial competition, and toxin–antitoxin-associated activity.
RTX toxin-associated system	*rtxB*, *rtxC*, *rtxD*	chromosome 1	RTX toxin activation and transport, contributing to cytotoxicity and host–cell interaction.
Cytolysin/hemolysin	*hlyA*	chromosome 2	Production of *Vibrio cholerae* cytolysin, associated with hemolytic and cytotoxic activity.

**Table 5:** Virulence-associated determinants identified in the genome of the *Vibrio parahaemolyticus* isolate (JCASQT010000000). Detected determinants are grouped according to functional category, genomic location, and predicted biological role.

Functional Category	Virulence-Associated Determinants	Genomic Location	Predicted Biological Role
Type III secretion system effector	VPA0450	Chromosome 1′	T3SS1-associated effector involved in host–cell interaction and cytotoxicity-associated mechanisms.
Exotoxin/Hemolysin	*tlh*	Chromosome 1′	Thermolabile hemolysin associated with membrane damage.
Motility/Chemotaxis	*fli*G, *che*Y, *che*W, *flg*B	Chromosome 2′	Flagellar motility and chemotaxis-associated genes, supporting environmental persistence, host interaction and colonization potential.
Adherence	*vpad*F, *mam*7	Chromosome 2′	Surface adhesins involved in bacterial attachment to host cells and early colonization.
Type III secretion system 1: regulatory and structural apparatus	*vxs*C, *vir*G, *exs*A, *exs*D, *vsc*B, *vsc*C, *vsc*D, *vsc*F, *vsc*G, *vsc*H, *vsc*I, *vsc*J, *vsc*K, *vsc*L, *vsc*U, *vsc*T, *vsc*S, *vsc*R, *vsc*Q, *vsc*P, *vsc*O, *vsc*N, *vsc*X, *vsc*Y, *vcr*D, *vcr*R, *vcr*G, *vcr*V, *vcr*H, *vec*A, *tye*A/*vcr*1, *syc*N/*vcr*2	Chromosome 2′	Components of the T3SS1 secretion machinery, regulators, chaperones and structural proteins involved in effector delivery into host cells.
Type III secretion system 1: secreted effectors/translocators	*vop*S, *vop*R, *vop*Q, *vop*N, *vop*B, *vop*D	Chromosome 2′	T3SS1-associated effector and translocator proteins involved in host–cell manipulation, cytotoxicity and delivery of virulence factors.

## Data Availability

The WGS data generated in this study have been deposited in the NCBI databases. The *Vibrio cholerae* genome is available under accession number JCASQS010000000 (BioProject PRJNA1470181), and the *Vibrio parahaemolyticus* genome under accession number JCASQT010000000 (BioProject PRJNA1470184). Further inquiries can be directed to the corresponding author.

## Abbreviations

AI-2	autoinducer-2
AMR	antimicrobial resistance
ANI	average nucleotide identity
APW	alkaline peptone water
ARG	antimicrobial resistance gene
AST	antimicrobial susceptibility testing
BHI	brain heart infusion
BIC	Bayesian Information Criterion
CARD	Comprehensive Antibiotic Resistance Database
CDS	coding sequence
COG	Clusters of Orthologous Groups
CTXφ	cholera toxin bacteriophage
DDBJ	DNA Data Bank of Japan
ENA	European Nucleotide Archive
EUCAST	European Committee on Antimicrobial Susceptibility Testing
GC	guanine–cytosine
GTFCC	Global Task Force on Cholera Control
KL	K-antigen locus
MATE	multidrug and toxic compound extrusion
MDR	multidrug resistance
MIC	minimum inhibitory concentration
MLST	multilocus sequence typing
MSHA	mannose-sensitive haemagglutinin
NCBI	National Center for Biotechnology Information
OCV	oral cholera vaccine
OL	O-antigen locus
ONT	Oxford Nanopore Technologies
RND	resistance-nodulation-division
RTX	repeats-in-toxin
ST	sequence type
T3SS1	type III secretion system 1
T6SS	type VI secretion system
TCP	toxin-coregulated pilus
TCBS	thiosulfate–citrate–bile salts–sucrose
UFBoot	ultrafast bootstrap
VFDB	Virulence Factor Database
WASH	water, sanitation, and hygiene
WGS	whole-genome sequencing
